# The active construction of the visual world

**DOI:** 10.1016/j.neuropsychologia.2017.08.003

**Published:** 2017-09

**Authors:** Thomas Parr, Karl J. Friston

**Affiliations:** Wellcome Trust Centre for Neuroimaging, Institute of Neurology, University College London, WC1N 3BG, UK

**Keywords:** Saccades, Attention, Scene construction, Bayesian, Salience, Memory, Hemineglect

## Abstract

What we see is fundamentally dependent on where we look. Despite this seemingly obvious statement, many accounts of the neurobiology underpinning visual perception fail to consider the active nature of how we sample our sensory world. This review offers an overview of the neurobiology of visual perception, which begins with the control of saccadic eye movements. Starting from here, we can follow the anatomy backwards, to try to understand the functional architecture of neuronal networks that support the interrogation of a visual scene. Many of the principles encountered in this exercise are equally applicable to other perceptual modalities. For example, the somatosensory system, like the visual system, requires the sampling of data through mobile receptive epithelia. Analysis of a somatosensory scene depends on what is palpated, in much the same way that visual analysis relies on what is foveated. The discussion here is structured around the anatomical systems involved in active vision and visual scene construction, but will use these systems to introduce some general theoretical considerations. We will additionally highlight points of contact between the biology and the pathophysiology that has been proposed to cause a clinical disorder of scene construction – spatial hemineglect.

## Highlights

• The neuroanatomy of active vision is reviewed.
• Saccadic eye movements are controlled by the oculomotor brainstem system.
• The brainstem system is influenced by the basal ganglia and cortical networks.
• Hemineglect can result from deficits in networks that select saccadic targets.
• Memory processes support the temporal continuity of visual experience.

## 1 Introduction

Although our experience of the visual world seems temporally and spatially continuous, the sensations we derive it from are not. Saccadic eye movements constitute a series of discrete fixations, interspersed by rapid movements. Little meaningful visual information is obtained as the eyes sweep from one fixation to the next (Bridgeman et al., 1975) and, at any moment, the proportion of the visual field from which any high resolution information is sampled is tiny. These observations, seemingly so contrary to perceptual experience, can be reconciled under the metaphor of perception as hypothesis testing Gregory, 1980, Friston et al., 2012a). By forming hypotheses about a continuous world, saccades can be deployed as experiments to adjudicate among alternatives. Note, however, that such experiments are necessarily designed in a biased, unscientific, manner (Bruineberg et al., 2016).

This view implies the perception of space is fundamentally tied to motor representations, as visual input at a point in space is the consequence of an experiment (saccade to that location) (Zimmermann and Lappe, 2016). This enactivist take on perceptual synthesis means that objects in the visual field become hypotheses or explanations for ‘what would I see if I looked there?’. In this review, we will describe the neuronal apparatus used to perform these experiments – and thereby implement active vision (Andreopoulos and Tsotsos, 2013, Mirza et al., 2016, Ognibene and Baldassarre, 2014, Wurtz et al., 2011). This functional anatomy consists of the brainstem network which gives rise to the nerves to the extraocular muscles. The superior colliculus is an important structure in this network, receiving input from both subcortical and cortical regions. Particular focus will be afforded structures that determine the choice of saccade target, and the mechanisms by which the data from previous saccades are combined, accumulated or assimilated to construct a seamless temporal experience (Marchetti, 2014). These mechanisms can fail in the damaged brain, and a common syndrome resulting from this failure is spatial hemineglect. Patients suffering from this fail to attend to one side (typically the left) of visual space (Halligan and Marshall, 1998). One manifestation of this attentional deficit is a decreased frequency of saccadic sampling in the neglected half of space relative to the other (Karnath and Rorden, 2012). This is despite intact early visual processing of stimuli on the neglected side, as evidenced by electrophysiology (Di Russo et al., 2007) and neuroimaging (Rees et al., 2000). We will try to address some of the links between the neurobiology of visual scene construction, and the consequences of its disruption. A number of theoretical concepts recur throughout this review. These include consideration of the mnemonic processes required for scene construction, the relationship between eye movements and attention, and the inferential (Bayesian) nature of these processes.

## 2 Brainstem oculomotor control

**Fig. 1:**
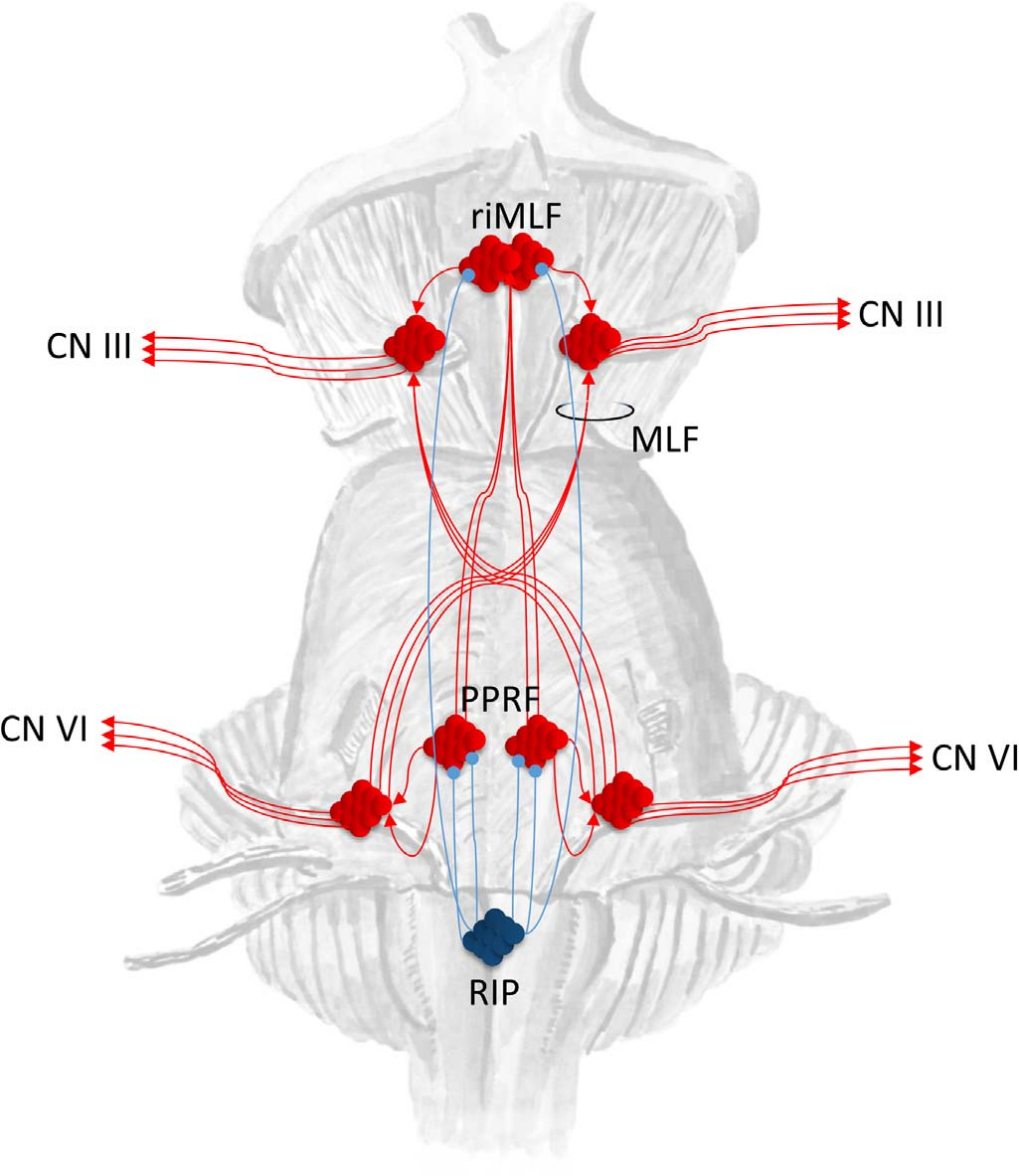
Brainstem control of saccadic movements This schematic shows some of the brainstem nuclei involved in the generation and control of saccadic eye movements. The paramedian pontine reticular formation (PPRF) is responsible for the generation of horizontal saccades, through its influence on the ipsilateral abducens nucleus, which gives rise to cranial nerve (CN) VI. A subset of neurons in the abducens nucleus projects to the contralateral oculomotor (CN III) nucleus in the midbrain, via the medial longitudinal fasciculus (MLF), ensuring conjugate eye movements occur. The PPRF additionally projects to the rostral interstitial nucleus of the MLF (riMLF), which generates vertical saccades. ‘Omnipause’ neurons in the nucleus raphe interpositus (RIP) synchronise the onset of vertical and horizontal components of saccades. The superior colliculus (not shown) influences both the PPRF and RIP. Excitatory connections are shown in red, while inhibitory connections are shown in blue. (For interpretation of the references to color in this figure legend, the reader is referred to the web version of this article.)

All forms of eye movement rely on the connections from the cranial nerve nuclei in the midbrain (CN III), the pons (CN IV), and the medulla (CN VI) to the extraocular muscles. Saccadic eye movements depend specifically upon the connections to these nuclei from the paramedian pontine reticular formation (PPRF) and the rostral interstitial nucleus of the medial longitudinal fasciculus (riMLF). The former generates horizontal saccades (Cohen et al., 1968, Henn, 1992), and the latter vertical (Büttner-Ennever and Büttner, 1978). Other important influences come from the vestibular system (Baker and Highstein, 1978), and the cerebellum (Berretta et al., 1993), but these will not be covered here. A subset of neurons within the PPRF monosynaptically target the ipsilateral abducens (CN VI) nucleus (Strassman et al., 1986). From the abducens nucleus, some neurons have axons which first decussate, then ascend as part of the MLF, to the oculomotor (CN III) nucleus in the midbrain (Sparks, 2002). The PPRF can use this pathway to initiate conjugate eye movements in the ipsilateral direction. An additional anatomical pathway allows the PPRF to influence the riMLF (Büttner-Ennever and Büttner, 1978), ensuring it can generate saccades with a vertical directional component (see Fig. 1 for a summary of this anatomy).

Saccadic movements are rapid movements that occur between short periods of fixation. In order to maintain fixation between saccades, PPRF ‘burst’ neurons are tonically inhibited by ‘omnipause’ neurons, located in the nucleus raphe interpositus (RIP) (Büttner-Ennever et al., 1988). These cells cease firing immediately before a burst of firing in the PPRF cells, but resume before the saccade is complete. ‘Omnipause’ neurons may have a role in synchronising different directional components of saccade generation, as the RIP also projects to the riMLF (Büttner-Ennever and Büttner, 1978). The electrophysiological correlates of the fixation and saccadic phases suggest the brain treats saccadic eye movements as a series of discrete events, consistent with the view that attentional processes are both serial and discrete (Buschman and Miller, 2010).

### 2.1 The superior colliculus

An important input to the PPRF, and the RIP, is the superior colliculus (Raybourn and Keller, 1977). This is a midbrain structure, found at the same level as the oculomotor (CN III) nucleus. The superior colliculus represents visual space according to several integrated topographic maps. Superficially, it contains a retinotopic map, making use of the input it receives directly from the optic nerve (Schiller and Stryker, 1972). Intermediate layers are thought to house a motor map, with each location corresponding to a potential saccadic target (Sparks, 1986). Deeper layers have maps that exhibit multisensory features, including somatosensation (Stein et al., 1989, Peck et al., 1993). Some accounts of collicular function propose that it contains a saliency map (Zelinsky and Bisley, 2015, Veale et al., 2017), and mediates attention to salient locations. Attention here refers to planned or performed eye movements leading to foveation of the ‘attended’ location. This is a distinct process to attention as ‘gain control’ (Hillyard et al., 1998, Feldman and Friston, 2010) of sensory streams (that does not necessarily depend upon oculomotor contingencies). The colliculus receives an input from cortical layer V (Fries, 1984). This layer specific input is shared with other structures with a role in salience computations, including the basal ganglia (Shipp, 2007) and the pulvinar nucleus of the thalamus (Shipp, 2003). It is encouraging that many of the areas implicated in attentional selection and salience conform to this laminar input pattern.

Neurons in the superior colliculus can be classified according to distinct electrophysiological profiles. Three broad categories of neurons are identifiable in this way. These are the collicular ‘burst’ neurons, the ‘fixation’ neurons, and the ‘build-up’ neurons (Ma et al., 1991, Munoz and Wurtz, 1995a). The first of the three are found more dorsally, while the latter two are more ventral within the colliculus. ‘Fixation’ neurons are active during fixation, and are found at the rostral pole of the colliculus. These synapse on the ‘omnipause’ neurons of the nucleus raphe interpositus (Gandhi and Keller, 1997), so that decreases in ‘fixation’ neuron activity causes a disinhibition of the PPRF ‘burst’ neurons, resulting in a saccade. The ‘burst’ neurons discharge immediately before a saccade, and the target location of the saccade corresponds to the location of these neurons in the colliculus. ‘Build-up’ neurons have a slowly increasing activity that terminates when a saccade occurs, although this activity is not always followed by a saccade. This observation is important in the context of the premotor theory of attention (Rizzolatti et al., 1987), as this theory suggests that covert attention may correspond to a planned saccade which does not take place. ‘Build-up’ neurons, as a population, have the interesting property that the activity across the population appears to travel as a ‘hill’ across the colliculus towards the rostral pole, which represents the foveal location (Munoz and Wurtz, 1995b).

The notion of a travelling ‘hill’ of excitation corresponds well to a set of theoretical constructs known as attractor networks. Representations of states which evolve in metric space have been extensively modelled using continuous attractor networks (Zhang et al., 2008). These rely on the assumption that a population code is used (Pouget et al., 2000), and there is good evidence to suggest that this is the case in the superior colliculus (Lee et al., 1988). One reason for emphasising this point is that, due to the serial nature of saccadic sampling, the apparent temporal continuity of visual experience requires explanation. The constraints placed upon a ‘hill’ of activity in a continuous attractor network mean that changing representation of one location in a metric space to another requires the transient representation of all intermediate locations. This enforces a form of memory, as the proximal future and past are heavily constrained by one another. This represents an imposition of prior beliefs on the interpretation of sensory data, providing a simple example of a form of Bayesian inference (Pouget et al., 2013).

If the superior colliculus is unilaterally damaged, or pharmacologically inactivated, the frequency of saccades to the contralateral side of space is reduced (Schiller et al., 1980, Schiller et al., 1987). However, in the presence of intact frontal eye fields, collicular ablation does not permanently prevent the generation of voluntary saccades (Albano and Wurtz, 1982). While this suggests that the frontal eye fields can make use of brainstem projections, which bypass the colliculus, reversible inactivation experiments indicate that the collicular route is the pathway used in structurally normal brains (Hikosaka and Wurtz, 1985a). The deficits following these pharmacological lesions resemble those observed in spatial hemineglect, as one side of space appears to be neglected by the lesioned animals, in terms of both saccadic sampling, and covert attention (Lovejoy and Krauzlis, 2010). The superior colliculus is rarely involved in lesions giving rise to neglect, but it is plausible that it is a component of the networks damaged in this syndrome – in the sense of a functional lesion or diaschisis (Price et al., 2001, Corbetta and Shulman, 2011). This brings us to consider the nature of the inputs to the colliculus.

## 3 The basal ganglia and eye movements

**Fig. 2:**
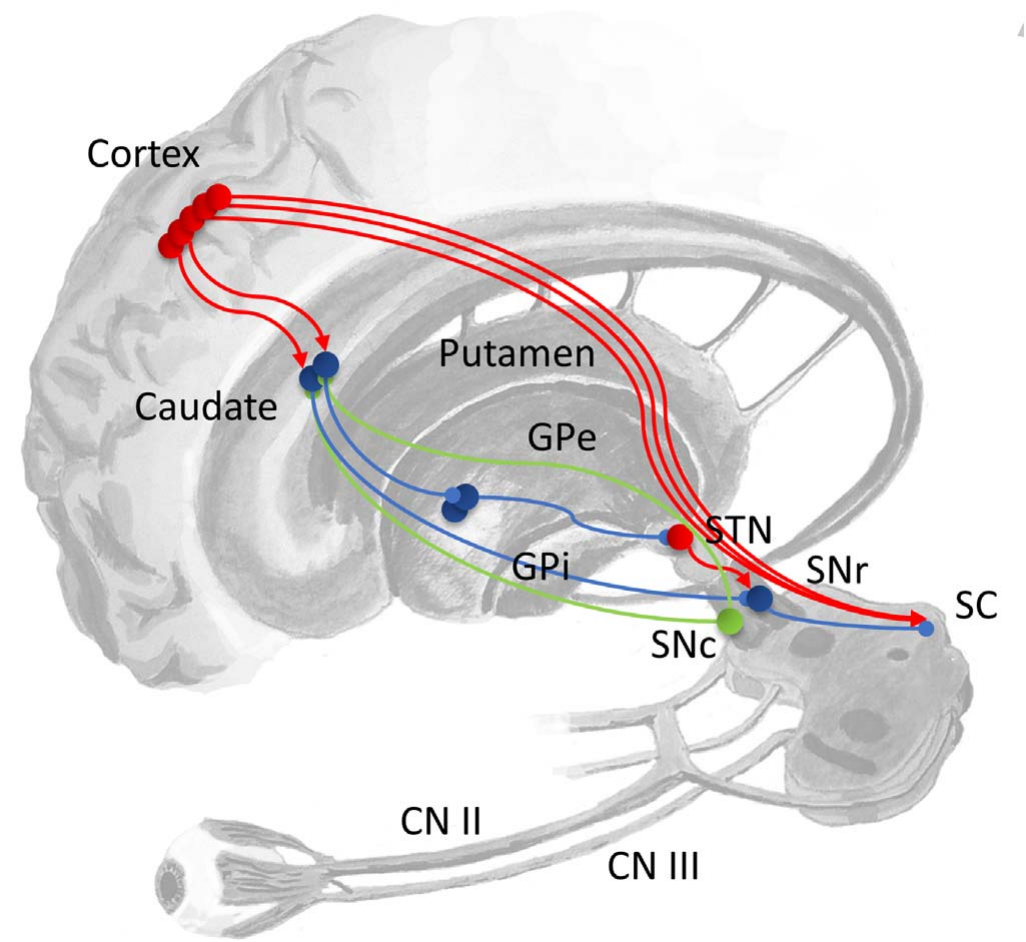
Contributions of the basal ganglia to eye movement control The superior colliculus is found in the midbrain at the level of the oculomotor nucleus, which gives rise to cranial nerve (CN) III. In addition to input from the optic nerve (CN II), it receives projections from the cortex and the substantia nigra pars reticulata (SNr). The SNr is a point of convergence between the ‘direct’ and ‘indirect’ pathways through the basal ganglia. The former is the path from the cortex, via the striatum (caudate and putamen), to the SNr or to the internal segment of the globus pallidus (GPi). The latter starts at the cortex, and also involves projections to the striatum. From here, the indirect pathway projects to the external segment of the globus pallidus (GPe), the subthalamic nucleus (STN), and then the SNr or GPi. The substantia nigra pars compacta (SNc), along with the ventral tegmental area (not shown), is a midbrain dopaminergic nucleus which provides a modulatory influence over the striatum. Excitatory connections are shown in red, inhibitory in blue, and modulatory in green. This schematic is based on descriptions by (Hikosaka et al., 2000). (For interpretation of the references to color in this figure legend, the reader is referred to the web version of this article.)

The substantia nigra pars reticulata (SNr) is an output nucleus of the basal ganglia located in the midbrain (Fig. 2). It has a direct inhibitory, GABAergic, connection to the superior colliculus (Hikosaka and Wurtz, 1983). This can be seen as a gate on the many direct cortical inputs to the colliculus, each of which identifies a different potential saccadic target. Consistent with this view is the observation that disruption of the SNr (Hikosaka and Wurtz, 1985b), or its projections to the colliculus (Hikosaka and Wurtz, 1985a), increases the frequency of spontaneous saccades. The SNr receives a glutamatergic input from the subthalamic nucleus, a component of the indirect and hyperdirect pathways through the basal ganglia (Nambu, 2004), and a GABAergic input from the D1 receptor expressing medium spiny neurons (MSNs) in the striatum, as part of the direct pathway. The striatum also contributes to the indirect pathway, as D2 receptor expressing MSNs inhibit the external part of the globus pallidus, thereby disinhibiting the subthalamic nucleus. The balance between the activity in the direct and indirect pathways is modulated by dopaminergic projections from the midbrain (Moss and Bolam, 2008), which act to bias this balance in favour of the direct pathway. Activity in the direct pathway disinhibits the targets of the basal ganglia output nuclei, while the indirect pathway increases this inhibition (Freeze et al., 2013).

### 3.1 The basal ganglia and memory

The role of the basal ganglia is now thought to extend far beyond the motor domain (Pennartz et al., 2009, Graybiel and Grafton, 2015). Theoretical studies have implicated the basal ganglia in the updating of working memory. This association can be motivated by appealing to the observation that caudate ablations can impair the performance of tasks involving a delay period – during which information must be retained in the absence of a stimulus (Battig et al., 1960). Additionally, some caudate neurons are known to have a greater activity when animals perform memory guided saccades (Hikosaka et al., 1989, Levy et al., 1997). Human studies back up these findings, as striatal dopamine synthesis capacity has been found to correlate with working memory span (Cools et al., 2008), and training in a working memory updating task appears to increase striatal dopamine release, as measured by greater ligand displacement from D2 receptors in a positron emission tomography (PET) study (Bäckman et al., 2011). Further to this, functional magnetic resonance imaging (fMRI) studies have demonstrated activation of basal ganglia components during updating of remembered stimulus arrays (Murty et al., 2011), and in the filtering of stimuli which are uninformative for a given task (McNab and Klingberg, 2008). The computational mechanisms proposed to explain the mnemonic functions of the basal ganglia typically assume representation of memories elsewhere – typically in the frontal cortex (Frank et al., 2001). The role of the basal ganglia, in these models, is to relieve inhibition of thalamocortical loops and allow updating of the cortical representations (Frank et al., 2001, Ebner et al., 2015). As dopamine modulates the balance between the disinhibitory direct and inhibitory indirect pathways, this neurotransmitter has been recruited to these models so that it can facilitate working memory updating.

A closely related approach (Gruber et al., 2006) makes use of dopaminergic projections to both the striatum and to the prefrontal cortex. This model suggests that the prefrontal cortex makes use of attractor networks which maintain a working memory. Such attractors are susceptible to drift, due to internal noise, and corruption by external signals representing distractor stimuli. Dopamine in the prefrontal cortex, according to the simulations arising from the model, protects against the latter, but not the former. Dopamine in the basal ganglia determines whether striatal MSNs are recruited when a new stimulus is presented. If so, they disinhibit neurons in the prefrontal cortex, allowing the new stimulus to drive a change in position of the activity ‘hill’. Robustness to internal noise has been incorporated into other models as a function of NMDA receptors on inhibitory interneurons in the prefrontal cortex (Murray et al., 2014a, Murray et al., 2014b). The degree to which these are activated could, in principle, be optimised; such that the degree of attractor drift mirrors the volatility of the stimulus along the dimension being represented. Behavioural experiments have validated some of the predictions arising from above model (Gruber et al., 2006). In particular, the memory corrupting effect of distractors is attenuated by manipulations thought to increase dopaminergic activity (Chumbley et al., 2008).

### 3.2 The basal ganglia and spatial hemineglect

Spatial neglect is often caused by cortical lesions. However, a number of subcortical regions have also been associated with the syndrome. An MRI study examined lesions in a number of patients with neglect, and compared the lesions to control subjects (Karnath et al., 2002). The putamen, pulvinar, and caudate nucleus were all found to be associated with neglect. These all communicate with cortical regions, such as the superior temporal gyrus, which, when damaged, can result in neglect. Changes in these regions have been observed following basal ganglia strokes which cause neglect (Karnath et al., 2005). In addition to these observational data, animal studies have demonstrated that a neglect-like syndrome can be induced through manipulations at the level of the striatum. Unilateral infusions of MPTP, which is toxic to dopaminergic axons, have been shown to bias memory guided (Kori et al., 1995) and spontaneous (Kato et al., 1995) saccades towards the ipsilateral visual field. As the dopaminergic input to the striatum is also affected in Parkinson’s disease, involvement of the basal ganglia plausibly explains the ‘directional hypokinesia’ component described in some forms of neglect (Mattingley et al., 1992). This is an impairment in initiating contralesional movements, more classically (but non-directionally) associated with Parkinson’s disease. In neglect patients who have anterior or subcortical lesions, ‘directional bradykinesia’ has additionally been observed.

## 4 Cortical connections and attention

**Fig. 3:**
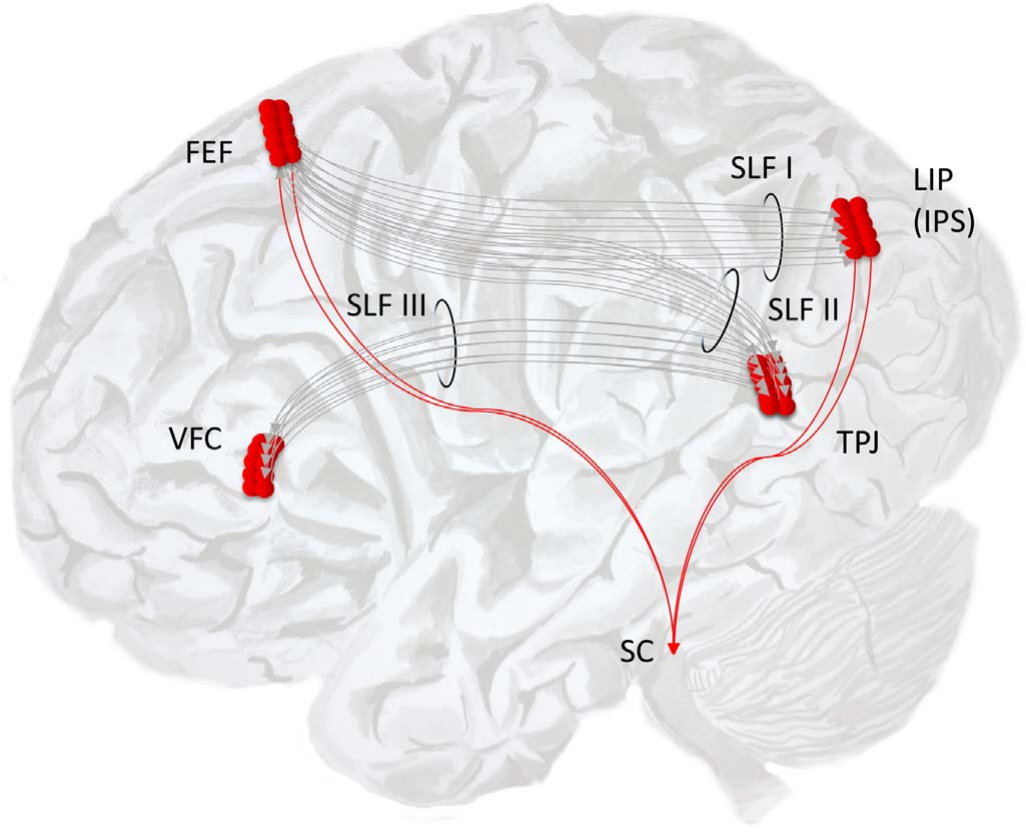
The dorsal and ventral attentional networks The dorsal and ventral networks each involve both frontal and parietal regions. The dorsal areas – including those in the region of the frontal eye fields (FEF), the lateral intraparietal (LIP) area and the intraparietal sulcus (IPS) – project to the superior colliculus (SC), suggesting a direct involvement of these areas in the control of eye movements. Note that these parietal areas are sometimes referred to as the parietal eye fields (Shipp, 2004). These areas are connected by the first branch of the superior longitudinal fasciculus (SLF I). The ventral network is made up of areas in the ventral frontal cortex (VFC) and areas close to the temporoparietal junction (TPJ). These are connected by the third branch of the SLF (SLF III). SLF II connects the parietal part of the ventral network to the frontal part of the dorsal network. This schematic is based on the descriptions in (Corbetta and Shulman, 2002) and in (Thiebaut de Schotten et al., 2011).

The cortical regions that project directly to the superior colliculus include both frontal (Künzle and Akert, 1977) and parietal (Gaymard et al., 2003) areas associated with the ‘dorsal attentional network’ (Corbetta et al., 2000, Szczepanski et al., 2013). This is a set of cortical regions which have been defined, using fMRI, on the basis of their signal changes during attentional tasks (Corbetta and Shulman, 2002). The activity in these areas is largely bilateral (Kastner et al., 1999, Hopfinger et al., 2000), but asymmetries have been found for some tasks (Corbetta et al., 2002, Szczepanski et al., 2010). Interhemispheric differences in regions of the dorsal network have also been elicited through causal manipulations, including transcranial magnetic stimulation (Szczepanski and Kastner, 2013), although the network as a whole was found to be approximately symmetrical. As might be expected for a region involved in directing eye movements, greater responses were found in the hemisphere contralateral to the visual field which was attended. These regions are connected by a white matter tract called the superior longitudinal fasciculus (SLF). The SLF is made up of three branches (Makris, Kennedy et al., 2004), and it is the first of these which connects the dorsal network of frontoparietal areas (Thiebaut de Schotten et al., 2011) (see Fig. 3). The other two branches connect the regions of the ‘ventral attention network’ to each other, and connect the dorsal and ventral networks to one another.

### 4.1 The premotor theory

The premotor theory of attention (Rizzolatti et al., 1987) draws evidence from these anatomical observations, as ‘attentional’ networks overlap substantially with those involved in eye movement control (Büchel et al., 1998, Corbetta et al., 1998, Nobre et al., 2000). The premise of this theory is that the allocation of (overt) attention to a given location is equivalent to making a saccade to that location. Attention can also be covertly directed to a location by planning a saccade to it, even if this saccade is not performed. The behavioural evidence for this theory comes from eye tracking studies in which the deployment of covert attention has been shown to systematically alter the trajectory of saccades (Sheliga et al., 1994, Sheliga et al., 1995). Psychophysical measures are consistent with this, as stimulus discrimination is enhanced at saccade target locations compared to other visual field locations (Deubel and Schneider, 1996). Further evidence comes from patients with palsies of the abducens (CN VI) nerve (see Fig. 1). These injuries result in an inability to abduct the eye on the affected side. In a detection task, consistent with the premotor theory, these patients do not show the reduced reaction time characteristic of covert attention when the stimulus is placed in a location which is impossible for them to perform a saccade to (Craighero et al., 2001). Physiological evidence in favour of the theory is compelling. By stimulating frontal eye field neurons in the monkey, it is possible to cause saccadic eye movements. Subthreshold stimulation of these same cells increases detection performance of stimuli presented at the saccadic target location of those neurons (Moore and Fallah, 2001). While not uncontroversial (Smith and Schenk, 2012), the premotor theory highlights the important relationship between attention and eye movements, and the anatomical structures common to both.

### 4.2 Active inference

The question of how a salient location is selected as a (covert or overt) saccadic target has stimulated much theoretical study. Bayesian frameworks have been extensively employed to address this question (Chikkerur et al., 2010), including definitions of salience, and surprise, in terms of information theoretic quantities (Itti and Koch, 2000, Itti and Baldi, 2006). More recently, this question has been formulated in terms of Active Inference (Friston et al., 2012a, Friston et al., 2012b; Mirza et al., 2016). This is a theory derived from the principle that adaptive (living) systems must minimise the dispersion of their states in order to continue to exist in a meaningful way (Friston et al., 2006). A consequence of this theory is that organisms should sample (e.g. by performing a saccade to) the parts of the sensory environment that resolve most uncertainty about the causes of their sensations. In order to select the locations that best serve this process, they are equipped with a probabilistic model of how sensory data is generated, which includes beliefs about their own actions (Friston et al., 2012a, b). This is used to generate predictions about the sensations they will encounter. By performing an approximate Bayesian inversion of this model, given sensory data, organisms are able to infer their own optimal policy (sequence of actions). Optimal in this context means the active sampling of sensations that afford the greatest reduction in uncertainty or, equivalently, the greatest information gain. This is also known as intrinsic value and, mathematically, is the expected Bayesian surprise that underwrites salience in the earlier formulations above (Itti and Koch, 2000, Itti and Baldi, 2006). A set of classical reflex arcs can then fulfil the predictions made under the implicit generative model (Friston et al., 2016a, Friston et al., 2016b). A key aspect of this Bayes optimal, epistemic, uncertainty resolving formulation implies that the best saccade is selected from representations of all possible saccades, according to their salience or epistemic value. In turn, this implies the existence of a salience map; where the epistemic values of all possible saccade locations are evaluated. This may provide a complementary perspective on the attractor dynamics discussed above as models of activity in the deep layers of the superior colliculus; namely, an encoding of salience.

### 4.3 Spatial hemineglect and attentional networks

In spatial hemineglect patients, cortical lesions can induce a lateral bias in the saccadic sampling of a scene. Typically, the frequency of saccades to the right side of space is increased, compared to the left. This appears to be related to the selection of saccadic targets, rather than an impairment in the production of saccades to the neglected hemifield (Bartolomeo and Chokron, 2002). Intuitively, one might expect the lesion sites to correspond to the dorsal frontal and parietal regions directly involved in saccadic control. However, although cortical lesions associated with neglect can occur in both frontal and parietal regions, they are typically more ventral than the frontal eye fields or the intraparietal sulcus (Corbetta and Shulman, 2002). A neglect-like syndrome can be elicited by lesioning the frontal eye fields (Latto and Cowey, 1971), but this is only temporary. Additionally, as noted above, the ‘dorsal attentional network’ is symmetrically distributed. This contrasts with the observation that spatial hemineglect is much more common following a right hemispheric lesion. While the behavioural correlates render it unlikely that cortically driven neglect precludes no dysfunction of the dorsal network, the above observations indicate that this is likely to be secondary to the disruption of other structures.

The lateral biasing of saccadic movements in neglect can be reconciled with the fact that the cortical inputs to the superior colliculus are often preserved. The more ventral frontoparietal regions which are associated with neglect overlap with the ‘ventral attentional network’ (Corbetta and Shulman, 2002, Corbetta and Shulman, 2011). In contrast to the dorsal network, the ventral network is more prominent in the right hemisphere, consistent with the greater frequency of spatial neglect following right hemispheric lesions. These regions are connected by the third branch of the SLF, which is known to have a greater volume in the right hemisphere (Thiebaut de Schotten et al., 2011). The ventral parietal regions of this network are connected to the frontal regions of the dorsal network by the second branch of the SLF. This means that the ventral network directly influences the cortical sites that project to the saccade generating areas of the brainstem.

The second branch of the SLF has been associated with some interesting lateralised behavioural correlates. In normal subjects, under certain conditions, a ‘pseudo-neglect’ can be elicited (Bowers and Heilman, 1980, Jewell and McCourt, 2000). This has been shown for a line bisection task, also used to assess hemineglect, in which a subject marks what they believe to be the midpoint of a horizontal line. While hemineglect patients typically mark to the right of the midline, small deviations to the left can occur in healthy subjects. The degree to which this ‘pseudo-neglect’ occurs is related to the volume of the right SLF II. The larger this is, the greater the leftward deviation (Thiebaut de Schotten et al., 2011). It has been proposed that neglect represents a disconnection syndrome, in which the frontoparietal interactions mediated by the SLF have been disrupted (Bartolomeo et al., 2007, He et al., 2007). This structurally motivated hypothesis complements the functionally motivated suggestion that an interaction between the dorsal and ventral networks is necessary for normal attentional function (Corbetta and Shulman, 2002). There is some evidence for this from lesion studies. For example, one study looking at lesion overlaps between patients found maximal subcortical overlaps in the SLF (Doricchi and Tomaiuolo, 2003). Case reports (Ciaraffa et al., 2013) endorse this finding, which is further strengthened by the observation that SLF II damage is a good predictor of hemineglect (Thiebaut de Schotten et al., 2014, Lunven et al., 2015). In addition to this, inactivation of the right SLF by electrical stimulation during surgery caused a temporary rightward deviation in the line bisection task (Thiebaut de Schotten et al., 2005).

### 4.4 Dorsal versus ventral

The distinction between the dorsal and ventral networks mirrors the distinction between the dorsal and ventral visual pathways (Goodale and Milner, 1992). These are often referred to as the ‘what’ and ‘where’ visual pathways, as the former appears to represent stimulus identity, while the latter represents stimulus location (Ungerleider and Haxby, 1994). Given that an object retains its identity, regardless of its position in space, the brain appears to have treated these as independent factors. In probabilistic inference, this is referred to as a ‘mean field approximation’ (Friston and Buzsáki, 2016). If the dorsal and ventral attention networks represent a similar factorisation, this could provide an intuitive explanation for the lateralisation of the latter network, and the symmetry of the former. Each hemisphere is thought to contain maps of the contralateral side of space (Wandell et al., 2007). It is unsurprising then that more dorsal regions, associated with the ‘where’ pathway, are relatively symmetrical. However, stimulus identity does not require representation in a specific location, due to the factorisation of these variables. As such, a unilateral representation is sufficient for the ‘what’ stream. This is consistent with clinical neuropsychological observations, as lesions to regions in the right ventral visual pathway are can give rise to disorders of object recognition (Warrington and James, 1967, Warrington and James, 1988, Warrington and Taylor, 1973), while the homologous regions on the left are more likely to be associated with difficulty naming objects (Kirshner, 2003). This could explain the lateralisation of the ventral network and, given its influence over the dorsal network, is consistent with the higher prevalence of spatial neglect among patients with right hemispheric lesions. The connection between the two networks would be mandated by the need to direct the eyes to different locations to resolve uncertainty about a stimulus or scene identity. According to this view, as the right SLF II connects the regions representing identity to those representing eye positions towards the left, damage to this structure impairs the selection of left sided saccadic targets. Note that a popular alternative explanation for this pathological asymmetry is that the right hemisphere represents both left and right sides of space, while the left represents only the right side (Mesulam, 1999). It is also plausible that lesions to the ventral network are more likely to extend to the right dorsal network than to contralateral regions. This explanation requires that hemineglect occurs when there are lesions of both networks.

## 5 Working memory and temporal continuity

As has been emphasised above, saccadic eye movements involve sampling of locations in a serial and discrete fashion. The frequency of spontaneous saccades is about 2–3 Hz (Büttner and Büttner-Ennever, 2006), but clearly we do not reset our beliefs about a visual scene at this frequency. In order to construct a temporally continuous representation of the visual world, it is clear that some form of short term memory must be involved, so that the information obtained at one fixation carries over – or is assimilated – into the next. Broadly, there are two mechanisms that allow the temporary storage of information in the brain. These are sustained neuronal activity (Goldman-Rakic, 1995), and short term changes in synaptic efficacy (Mongillo et al., 2008). In Bayesian approaches to understanding brain function, these two mechanisms correspond inference and learning respectively; namely, updating beliefs (approximate posterior distributions) about hidden states of the world, and parameters (generative model) that describe the probabilistic relationships between hidden states (Friston et al., 2016a, Friston et al., 2016b).

### 5.1 Memory as sustained neuronal activity

Sustained neuronal activity has been extensively studied in the context of ‘delay-period’ activity (Goldman-Rakic, 1995). This is the increase in firing rate observed in some neurons, which persists even after the stimulus that evoked the increase is no longer present. ‘Delay-period’ working memory tasks during single unit recordings have been used to demonstrate this phenomenon (Funahashi, 2015). An example of such a task is an oculomotor delay task, in which an animal fixates a location on a screen. A stimulus is presented which indicates a saccadic target. During a delay, in which no stimulus is present, the animal must remember the target location. When instructed, they should perform a saccade to that location. From the presentation of the stimulus, until the performance of the saccade, neurons in the principal sulcus of the prefrontal cortex remain persistently active (Funahashi et al., 1989). Among these neurons, many are tuned to the eventual saccade direction. Other parts of the frontal cortex have been shown to contain populations of neurons that exhibit similar properties for other planned actions (Cisek and Kalaska, 2005). The relationship between these forms of memory and planned actions have prompted some authors (Hikosaka et al., 2000, Frank et al., 2001) to suggest that the raison d′être of working memory is in evaluating future actions. This complements work on decision processes in the field of artificial intelligence (Kaelbling et al., 1998), in which memory serves a similar purpose. There is an attractive circularity to the notion that the temporal continuity of visual experience is due to the use of memories from past saccades to evaluate potential future saccades.

Single unit recordings have demonstrated that there are neurons with responses limited to the duration of a stimulus presentation (Hubel and Wiesel, 1959), and also those which have responses that transcend this time scale (Funahashi et al., 1989). This speaks to a temporal hierarchy (Hasson et al., 2008, Kiebel et al., 2008, Murray et al., 2014a, Murray et al., 2014b Cocchi et al., 2016) in the brain, with different neurons representing different rates of environmental change. Temporal responses in different areas of the brain have been shown (Hasson et al., 2015, Hasson et al., 2008, Honey et al., 2012, Murray et al., 2014a, Murray et al., 2014b) to map closely to the hierarchical structure of the cortex as derived from studies of laminar connectivity (Zeki and Shipp, 1988, Felleman and Van Essen, 1991). This is consistent with the idea that the brain contains a hierarchical generative model (Friston, 2008) of a temporally structured environment, and allows for slowly changing contexts to inform the evolution of states which change over a faster time scale. Under this view, working memory, in the form of persistent neuronal activity, corresponds to a process of evidence accumulation over multiple timescales.

As mentioned above in the context of the superior colliculus, sustained activity patterns have been extensively modelled using continuous attractor networks. Working memory has not escaped this treatment (Compte, 2006, Wimmer et al., 2014). While many accounts of working memory focus on prefrontal regions, such networks have been used to model activity in many different brain regions, including those for brainstem oculomotor control (Seung, 1998), navigational regions (Redish et al., 1996, Zhang, 1996), and motor planning (Georgopoulos et al., 1982, Lukashin et al., 1996). Given the computational nature of these architectures, all could be described as implementing a form of working memory. All involve a sustained representation, which is updated as new observations are made. However, these memories are have different temporal properties, depending on the rate of change of what they represent, and so may not be sustained over the time course associated with the classical notion of working memory. Notably, it is areas considered high in the anatomical (and consequently temporal) hierarchy (Felleman and Van Essen, 1991), such as the dorsolateral prefrontal cortex (Kojima et al., 1982, Goldman‐Rakic, 1987), which are often thought to perform working memory functions.

### 5.2 Memory as short term plasticity

**Fig. 4:**
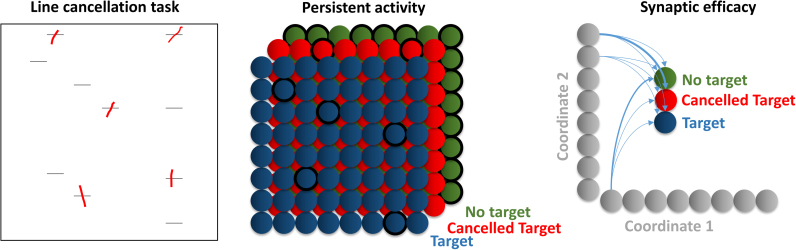
Mechanisms of memory *On the left,* an example of a line cancellation task is shown. The subject is presented with a sheet of paper with a set of horizontal lines, and is asked to cancel (red marks) each of these lines. *The middle panel* shows the set of 192 neurons which would be required to represent the subject’s beliefs about where the lines are, and whether they have cancelled them, if the memory of previously visited locations were stored in terms of persistent activity in a neuronal population. The currently active neurons are represented by a black outline. *The panel on the right* shows a more efficient way to represent this information, in terms of a mapping from a representation of space to representations of each of the possible observations that could be made on visiting a particular location. Clearly it is more efficient to make use of synaptic efficacy when storing temporary, high dimensional, memories. In short, synaptic efficacy represents probabilistic mappings (i.e., ‘if I were to look there, I would see that’) as opposed to beliefs about the current state of the world (i.e., ‘I am looking there’ or ‘seeing that’) encoded by synaptic activity.

For some situations requiring working memory, persistent activation of neurons is an inefficient way to store temporary information. This is due to the number of dimensions required for some memories, and the metabolic constraints (Lennie, 2003) on the number of neurons required to represent these. To build some intuition for this point, consider the example (shown graphically in Fig. 4) of a cancellation task. Variants of these tasks are frequently used both clinically (Albert, 1973, Fullerton et al., 1986) and experimentally (Husain et al., 2001, Malhotra et al., 2004, Mannan et al., 2005) to assess spatial neglect. Subjects are shown an array of targets, and are asked to cancel each target once, and only once. Cancellation may involve marking the target with a pencil, or clicking on it in a computer display. In the latter set up, there need not be a visible marker alerting the subject that they have previously cancelled it. Despite this, there is a relatively low rate of re-cancellation of a stimulus in healthy subjects (Mannan et al., 2005), showing that cancelled locations are remembered. If this task were performed using a set of possible locations on an 8 × 8 grid, there would be 64 possible target locations. For each of these, there are 3 possible states: no target, target, and cancelled target. To be able to represent beliefs about the state at each location as persistent activity in populations of neurons, it would be necessary to employ 64 × 3 = 192 computational units, and to maintain activity patterns across all of these simultaneously. In many natural scenes, the number of locations, and possible stimuli at each location, is clearly much greater than this, and would require huge numbers of neurons if remembered in this manner.

Contrast this with a memory system in which information is stored in the interactions between different neurons (i.e. synaptically). In this case, it is only necessary to employ 3 computational units to represent the state at each location. Each location unit can then represent its current state as an interaction between itself and the three alternative states. For example, on viewing a target for the first time, the synapses between the unit representing the location and that representing the presence of a target can be potentiated. This reduces the need for 192 neuronal populations to 67; a number which can be further reduced to 19 using a factorised representation of location (i.e. a coordinate system) in place of explicit representations of each location. This simple example demonstrates that, while low dimensional memories can be stored as persistent activity, synaptic updates are a much more efficient way to store higher dimensional representations. This might explain why some working memory tasks have failed to show a clear relationship between working memory deficits and re-cancellation rates in neglect patients (Wansard et al., 2014). There may be impairment in (short-term) synaptic plasticity, which would not be detected by probing with a delay-period type task. As highlighted by one of our reviewers, a complementary perspective on this issue (Jewell and McCourt, 2000) is afforded by the notion of acquired scanning patterns and related sensorimotor coupling (Chokron et al., 1998, Speedie et al., 2002). In other words, the natural biases engrained into active perception, through synergy with the environment (Verschure et al., 2003).

Short term plasticity may be due to several mechanisms, but calcium dependent processes clearly play a substantial role. In a presynaptic neuron, an increase in calcium ion concentration, as a result of an action potential, triggers vesicular release. With repeated action potentials, intracellular calcium buffers can become saturated (Blatow et al., 2003, Deng and Klyachko, 2011), ensuring that the increase in calcium at the next action potential will be greater. This means that the synapse is temporarily potentiated. Pre and postsynaptic mechanisms have been used to explain the opposite phenomenon, in which there is a temporary depression of the synapse. Changes in plasticity over very short time scales, such as these, have been described in neurons in the prefrontal cortex (Hempel et al., 2000, Wang et al., 2006). Computational studies (Mongillo et al., 2008, Barak et al., 2010) have demonstrated that dynamics such as these could account for some working memory phenomena.

Spatial neglect provides some clues as to the anatomical regions that may be involved in this kind of short term plasticity for spatial memories (Mannan et al., 2005). For patients with lesions of the intraparietal sulcus, the probability of re-cancellation of a target increases with time. In contrast, lesions of the inferior frontal regions give a constant increased re-cancellation probability. Although both regions are related to the attentional networks, these results suggest distinct mechanisms of neglect following each lesion. The former appears to be memory dependent, while the latter does not. This hints at the importance of axons in the region of intraparietal sulcus. These connections could furnish the candidate synapses that store spatial memories through short term plastic changes. Consistent with this, patients with neglect who have a more severe spatial working memory deficit have been reported to have parietal white matter lesions not found in those with who have neglect but relatively intact spatial working memory (Malhotra et al., 2005).

## 6 Conclusion

The neuroanatomical system which supports the interrogation of a visual scene includes a complex network of brainstem areas under the influence of cortical and subcortical structures. Damage to almost any component of this system can cause a neglect syndrome, emphasising their important roles in visual experience. The mnemonic properties of many of these components have been highlighted, as these allow information from the past to be integrated into representations of the present and future. In other words, posterior beliefs following one observation become prior beliefs about the causes of the next. The updating of this form of working memory on the basis of new observations is necessarily a Bayesian (belief updating) process, likely involving a factorisation of variables, such that ‘what’ and ‘where’ are represented independently. This is consistent with the dorsal and ventral streams hypothesis, and the anatomy of the attentional networks, which provide a cortical influence over eye movements. In doing so, hypotheses derived from past experience are combined with new sensory data to construct visual percepts.

## Disclosure statement

The authors have no disclosures or conflict of interest.
